# European radiation oncology after one year of COVID-19 pandemic

**DOI:** 10.1016/j.ctro.2021.03.011

**Published:** 2021-04-16

**Authors:** Ben J. Slotman, Valerie Cremades, Anna M. Kirby, Umberto Ricardi

**Affiliations:** a Department of Radiation Oncology, Amsterdam University Medical Centers, Amsterdam, the Netherlands; b European SocieTy of Radiation Oncology, Brussels, Belgium; c Department of Radiation Oncology, Royal Marsden Hospital & Institute of Cancer Research, Sutton, UK; d Department of Oncology, University of Turin, Turin, Italy

## Abstract

• Survey of European radiotherapy department heads after 1 year of Covid-pandemic.
• A decrease in patient volume was reported in 53%.
• Enrollment in clinical studies was decreased in 58%.
• Concerns were expressed related to well-being of staff in 76% of the departments.

## Introduction

The COVID-19 pandemic had an enormous impact on our health care system, including the care for cancer patients [1], [2]. These patients are a vulnerable group due to their compromised clinical condition and the urgency to initiate and not interrupt the treatment. Shortly after the first wave of the pandemic, we reported on its effect on radiotherapy care throughout Europe and beyond [3], [4] based on a survey carried out in conjunction with the American Society for Radiation Oncology (ASTRO). In addition, numerous articles and editorials have been published on the effect of the pandemic on cancer treatment and on measures for specific disease sites or treatment modalities [5], [6], [7], [8].

The European SocieTy for Radiotherapy and Oncology (ESTRO) was more recently invited by the American Society for Radiation Oncology (ASTRO) to re-survey their members using ASTRO’s updated questionnaire, with the aim of better understanding the impact of the pandemic on health care provision in radiation oncology.

## Materials and methods

The questionnaire, slightly modified to be used by ESTRO (see supplementary materials), was sent on February 12, 2021, to a total of 500 ESTRO members, 474 of whom were registered as head of a radiation oncology (RO) department in Europe. In addition, it was sent to 26 representatives of other departments with no registered head. Reminder were sent after 7 and 12 days and the closure date was February 26, 2021.

## Results

After 14 days, 104 (nearly) completed questionnaires were received (response rate 21%) from 28 different countries. Most responses were from Italy (18; 17%), Belgium (14; 13%), Spain (11; 11%), Germany (7; 7%), Switzerland (7; 7%) and Romania (6; 6%). The remaining 41 each represented less than 5% of all responses and were from the 22 other countries.

The responding departments treat a median of 1,300 new cancer cases per year (range: 300–7,000), with a median number of patients under treatment of 85 per day (range 10–550). The staffing levels were at a median of 10 FTE (full time equivalent) radiation oncologists (range: 1–52) and 17 FTE radiation technologists (range: 3–140). All departments were operational. About half (51) of the centers delivered radiotherapy at more than one location. One center closed a satellite location due to shortage of staff. In all other centers, all locations remained open.

A decline in cancer screening for one or more groups of patients was seen in 69% of the centers and 71% noticed that patients were presenting with more advanced disease than before the pandemic.

Nine per cent of the departments noticed an increase in referrals in 2020 compared to 2019, with a median increase of 10% (range 2–13%). However, 38% noticed no change and 53% reported a decrease in patient volume. The median decrease was 10% (range 2–42%). A 1–10% decrease in practice revenue over 2020 compared to 2019 was reported by 47 centers (50%), a 11–20% decrease in 20 centers (21%) a 21–30% decrease in 8 (9%) and a more than 30% decrease in 7 centers (7%). Twelve centers (13%) reported an increase in practice revenue over 2020. Causes for the reduction in volume were delays/deferrals for certain tumor sites in 38%, reduced referral rates in 34% and shortage of staff in 3%.

In 23% of the departments, radiotherapy for some indications was deferred. The most frequently delayed indications were: low-risk prostate cancer (8%), non-malignant diseases (7%) and early stage breast cancer (3%).

The following measures were in place for the staff at the time of the second questionnaire: Routine use of masks (100%), testing of staff with symptoms (98%), increased sterilization of the clinic (90%), social distancing (78%), use of gloves (63%), face shields (40%) and/or gowns (32%) for treatments and procedures, screening prior to each shift (54%) and staggered shift scheduling (26%). Measures for patients included the routine use of masks (96%), social distancing (92%), allowing no visitors (76%) and screening at the front door (75%). Table 1 shows a comparison with measures in place during the start of the pandemic.

**Table 1:** Important measures in place during first (May 2020) and second (February 2021) survey.

	May 2020[3]	February 2021
Deffered treatment	58%	23%
Telemedicine	78%	65%
Routinely using masks by personnel	88%	100%
Staggered shift scheduling	58%	26%
Social distancing	88%	78%
Patient screening for symptoms at front door	82%	75%
No visitors allowed	88%	76%

Telemedicine was used in 65% of the departments. It was used for new patients in 12%, clinical assessment of patients under treatment in 11% of the departments and for routine follow-up in 64%.

Interruptions of treatments were reported by 50 departments (55%). This was due to Covid-19 illness of the patient (50/50 departments), patient caregiver quarantine protocol or illness (18/50 departments) and/or limited hospital capacity for brachytherapy procedures (6/50 departments). The majority of departments (67; 73%) created specific procedures for treatment of Covid-19 positive patients to continue treatments without interruptions.

Vaccination of personnel has started in 54% of the centers (49). In 67% of the centers, all staff physicians had received at least the first vaccine dose. These numbers were 60% for nursing staff, 58% for physicists, 57% for resident physicians, 55% for RTT’s, 52% for dosimetrists and 46% for administrative staff. Access to the vaccine was reported as a barrier to vaccination in 54% of the centers. Distrust in and/or unwillingness of staff to receive the vaccine was a barrier in one or more persons in 23% of the centers (21).

A permanent or transitory reduction in staff was observed in 70% of the departments. Main reasons included Covid-19 illness of staff in 50% of the departments, impact of the COVID-19 pandemic on family care responsibilities in 43%, staff transfer to other clinical areas in 16% and reduced number of patients visits in 4%. Covid-19 illness in some or more residents was seen in 62% of the departments, in 78% of oncologists, in 76% of radiotherapy technologists (RTT’s) and nursing staff, in 44–49% of physicists, dosimetrists and administrative staff.

Shortages of personal protective equipment during the pandemic were reported by 29% of the departments, of medical hand sanitizer and of nasopharyngeal swabs for Covid-19 specimen collection in 12% each.

Clinical and/or translational research is conducted in 68% of the centers (65). Fifty-nine centers had open clinical trials and seven of them (12%) noticed an increase in enrollment, whereas 58% noticed a decrease and 31% noticed no change in the enrolled number of patients. New Covid-19 research concepts were added to the research portfolio in 45% of the centers.

The department heads expressed concerns related to the well-being of health professionals (76%), creating flexible work arrangements for staff with family needs (66%), burnout of health professionals (61%), and work/life balance of health professionals (54%).

## Discussion

Compared to the questionnaire in 2020 [3], the response to this second questionnaire was lower (21 vs. 28%), with especially fewer responses from The Netherlands and the United Kingdom. This is a potential bias that complicates direct comparison. Our study did not evaluate the introduction of more hypofractionated radiotherapy schemes. A population-based study in the United Kingdom recently demonstrated a decline in radiotherapy activity, but also an increased use of hypofractionation for various indication and potentially a shift from surgical treatments to radiotherapy [9].

During the first year of the COVID-19 pandemic radiotherapy departments have tried to continue their care as much as possible and to deliver timely and uninterrupted courses to all patients. A decrease in referrals was seen in about half of the centers. This decrease was mostly caused by their own decision to delay or defer treatment and a reduced referral rate. Surprisingly, 10% of the centers saw some increase in patient volume.

Compared to the first questionnaire in May 2020 [3], reduction of staff was seen more often (70 vs. 57%), mainly due to Covid-disease and family care. Most departments acknowledge the effect of the pandemic on the workload, work-life balance and the risk of burnout. Protective measures for staff and patients were maintained during the pandemic. The use of telemedicine was somewhat reduced compared to 2020, with 65% instead of 78% of the centers using telemedicine, and mainly used for follow-up visits.

This questionnaire was filled in around 2 months after the start of vaccination in most countries. Just over half of the centres had started vaccination of personnel, and depending on the discipline, 45–67% of the personnel in these departments had received their first dose. Access to the vaccine was still an important problem in 54% of the centers, while hesitance to take the vaccine in one or more employees was an issue in 23% of the centers.

The pandemic also had an effect on clinical and translational research with fewer patients entered into trials. It is also interesting to see that 45% of the centers mention that they started new research related to Covid-19.

In conclusion, radiotherapy departments throughout Europe managed to continue the treatment of the patients. Around 70% of the centers already reported a decline in screening and noticed an increase in patients presenting with more advanced disease for one or more indications. The current back-log in referred patients will probably lead to a future increase in radiotherapy referrals. In addition, the decrease in number of treated patients could lead to future presentations with more advanced disease.

## Declaration of Competing Interest

The authors declare that they have no known competing financial interests or personal relationships that could have appeared to influence the work reported in this paper.
