# Supplementary material for: European radiation oncology after one year of COVID-19 pandemic
Source: Clin Transl Radiat Oncol. 2021 Apr 16;28:141–3. doi: 10.1016/j.ctro.2021.03.011 (PMC8049845; doi:10.1016/j.ctro.2021.03.011)
Supplement: Supplementary Data 1 [file mmc1.docx]

**2021 ESTRO Practice Response Survey (COVID-19) - Follow-up**

Q1 In which country are you based?

Q2 How many radiation oncologists are in your practice?

Q3 How many RTTs (radiation therapists) are in your practice?

Q4 Do you provide radiation oncology care/services at multiple facilities?

Yes

No

Q5 Approximately how many new cancer cases did your practice treat in 2020?

Q6 Is your practice currently providing radiation therapy treatment services to patients?

Yes

No

Q7 If you are providing radiation oncology care/services at multiple

facilities, at any point since the COVID-19 pandemic started, have you closed any satellite locations?

Yes, we have closed at least 1 satellite location

No, all locations are currently open

Not applicable since we only have one facility

Q8 You mentioned that you closed at least 1 satellite location, please tell us why?

Insufficient patient volume

Shortage of staff

Other (please specify)

Q9 How is your practice scheduling new patient visits at this time?

No change in scheduling new patient visits due to COVID-19 pandemic

Deferring some but not all new patient visits (i.e. benign disease)

Not accepting any new patients at this time

Q10 Is your practice providing telemedicine visits? (Select all that apply)

Yes, for new patient consults

Yes, for clinician assessments of patients currently on treatment

Yes, for routine visits for patients for follow-up

No

Q11 How many patients do you currently have on treatment at your practice (patients treated per day)?

Q12 Physicians at my practice are noticing that some groups of patients are not receiving cancer screenings.

True

False

Q13 Patients are presenting to my practice with more advanced disease compared to prior to the COVID-19 pandemic.

True

False

Q14 In 2020, did the COVID-19 pandemic cause a change in patient

volume at your practice compared to 2019?

Increase in patient volume

No change in patient volume

Decrease in patient volume

Q15 You mentioned that the COVID-19 pandemic led to a decline in

patient volume, what was this due to? (Select all that apply)

Patient volume decrease due to delays/deferrals of treatment for certain diseases

Referrals slowing down

Shortage of staff

Other (please specify)

Q16 You mentioned that the COVID-19 pandemic caused an increase in patient volume at your practice, please tell us how much your patient volume has increased (%) compared to the typical volume?

Q17 You mentioned that the COVID-19 pandemic caused a decrease in patient volume at your practice, please tell us how much your patient volume has decreased (%) compared to the typical volume?

Q18 What percentage would you estimate has practice revenue in 2020 decreased compared to 2019 due to the COVID-19 pandemic?

1-10% decrease

11-20% decrease

21-30% decrease

31-40% decrease

41-50% decrease

51% or more decrease

Revenue will increase in my practice

Q19 Is your practice currently delaying treatment of any disease sites?

Yes

No

Q20 What disease sites/treatments are you currently delaying? (Select all that apply)

Early Stage Breast

Locally Advanced Breast

Small Cell Lung

NSCLC Early Stage SBRT

NSCLC Locally Advanced

Early Stage Definitive HN

Locally Advanced Definitive HN

Post Operative Adjuvant HN

CNS - Low Grade Glioma

CNS - High Grade Glioma

CNS - GBM

GI - Esophageal/Gastric

GI-Pancreas

GI-Liver

GI-Rectal

GI-Anal

Prostate - Low Risk

Prostate - Intermediate Risk

Prostate - High Risk

Bladder

Sarcoma

GYN-Cervical

GYN-Uterine

GYN-Vagina/Vulva

Palliative Non-Emergent

Palliative Emergent

Oligometastatic SBRT

Cutaneous: Melanoma

Cutaneous: Non-Melanoma

Non-malignant conditions (i.e. acoustic neuroma, AVM)

Lymphomas and Leukemia

Pediatric high grade CNS

Pediatric low grade CNS

Pediatric solid tumor

Pediatric leukemia/lymphoma

Q21 For each of the measures below, what is your current practice for staff in response to the COVID-19 pandemic?

Screening of staff prior to each shift (i.e. fever, symptoms)

Staff who exhibit symptoms are tested for COVID-19

Staff routinely wearing masks

Staff wearing face shields for treatments/procedures

Staff in gloves for treatments/procedures beyond routine precautions

Staff in gowns for treatments/procedures

Staggered shift scheduling of staff

Increased sterilization/cleaning of clinic

Social distancing in clinic (6 feet, single room occupancy)

Q22 For each of the measures below, what is your current practice for patients in response to the COVID-19 pandemic?

Screening of patients/visitors at front door (hospital, center, and/or department)

No visitors allowed

Patients routinely wearing masks

Social distancing in clinic (6 feet, single room occupancy)

Q23 Patients in my practice have experienced social or financial hardship during their radiotherapy that has been worsened by the COVID-19 pandemic.

True

False

Q24 Have patients at your practice who started radiation therapy

experienced any treatment interruption during their treatment course due to the COVID-19 pandemic?

Yes

No

Q25 Please indicate the types of interruptions your patients experienced? (Select all that apply)

Patient COVID illness has caused patients to interrupt therapy mid-treatment

Limited hospital capacity has caused delays or interruptions for brachytherapy procedures (i.e. T&O implants for cervical cancer, LDR boost for prostate cancer)

Patient caregiver quarantine protocol and illness has caused patients to interrupt treatment

Other (please specify)

Q26 Has your practice created a specific procedure (i.e. treatment at end of day with staff in PPE, treatment at specific satellite center) to continue radiation treatments without interruption for clinically stable COVID19+ patients?

Yes

No

Q27 As of today, please indicate of those who are willing, who has been vaccinated at your practice (i.e. at least received the initial dose).

Residents

Experienced Physicians

Medical Physicists

Dosimetrists

Nursing staff

RTTs

Administrative staff

Patients

Q28 Access to the vaccine has been a barrier to the COVID-19

vaccination in my practice.

True

False

Q29 Distrust in and/or unwillingness to receive the COVID-19 vaccine has been a barrier to vaccination of staff (e.g. physicians, nurses & RTTs) in my practice.

True

False

Q30 Distrust in and/or unwillingness to receive the COVID-19 vaccine has been a barrier to vaccination of patients in my practice.

True

False

Q31 Has your practice had a shortage in staff due to any of the following? (Select all that apply)

Staff COVID-19 illness

Staff availability due to impact of COVID-19 pandemic on family care responsibilities

Reduction in staffing due to reduced patient visits

Staff transfer to other clinical areas

No

Q32 You mentioned that there was a shortage in staff due to staff COVID-19 illness; please indicate how various roles have been impacted?

Residents

Experienced Physicians

Medical Physicists

Dosimetrists

Nursing staff

RTTs

Administrative staff

Q33 Has the COVID-19 pandemic impacted hiring practices in your

department?

Yes

No

Q34 Has your practice experienced shortages or limited access to any of the following resources during the COVID-19 pandemic? (Select all that apply)

Nasopharyngeal Swabs for COVID-19 Specimen Collection

Medical Hand Sanitizer

Personal Protective Equipment (e.g. N95 masks, surgical masks, other masks, gowns, gloves, etc.)

No, our practice is not experiencing any of the above shortages

Q35 Does your practice conduct basic, clinical and/or translational

research?

True

False

Q36 In 2020, did the number of patients your practice enrolled in clinical trials increase, stay the same or decrease?

Increase

Stay the same

Decrease

My practice does not enroll patients in clinical trials

Q37 Were new COVID research concepts added to your basic research portfolio?

Yes

No

Q38 In your role as a leader, are you concerned for your colleagues for any of the following due to stress associated with COVID? (Select all that apply)

Well-being of health professionals

Burnout of health professionals

Creating flexible work arrangements for staff with family needs

Work/life balance of health professionals

Increased complexity or severity of cases

Staying current with new clinical trends/techniques

Other (please specify)

Q39 What was the date your practice last provided radiation therapy treatment services? (mm/dd/yyyy)
